# Lipoleiomyomas of the Uterine Cervix: A New Series including the First Recurrent Case and the First Systematic Literature Review

**DOI:** 10.3390/jpm12111852

**Published:** 2022-11-06

**Authors:** Andrea Palicelli, Laura Ardighieri, Giuseppe Broggi, Rosario Caltabiano, Beatrice Melli, Maria Carolina Gelli, Magda Zanelli, Maria Paola Bonasoni, Aleksandra Asaturova, Maurizio Zizzo, Lorenzo Aguzzoli, Ruggero Baraldi, Vincenzo Dario Mandato

**Affiliations:** 1 Pathology Unit, Azienda USL–IRCCS di Reggio Emilia, 42123 Reggio Emilia, Italy; 2 Pathology Unit, ASST Spedali Civili di Brescia, 25123 Brescia, Italy; 3 Department of Medical and Surgical Sciences and Advanced Technologies “G.F. Ingrassia” Anatomic Pathology, University of Catania, 95123 Catania, Italy; 4 Molecular Pathology Unit, Azienda USL-IRCCS di Reggio Emilia, 42123 Reggio Emilia, Italy; 5 FSBI “National Medical Research Centre for Obstetrics, Gynecology and Perinatology Named after Academician V.I.Kulakov” of the Ministry of Health of the Russian Federation, 117513 Moscow, Russia; 6 Surgical Oncology Unit, Azienda USL- IRCCS di Reggio Emilia, 42123 Reggio Emilia, Italy; 7 Unit of Surgical Gynecologic Oncology, Azienda USL-IRCCS di Reggio Emilia, 42123 Reggio Emilia, Italy; 8 Unit of Obstetrics and Gynecology, Guastalla Hospital, Azienda USL-IRCCS di Reggio Emilia, 42123 Reggio Emilia, Italy; 9 Unit of Obstetrics and Gynecology, Azienda USL-IRCCS di Reggio Emilia, 42123 Reggio Emilia, Italy

**Keywords:** lipoleiomyoma, cervix, uterus, gynecological, histopathology, smooth muscle tumor, leiomyoma, review, recurrence, prolapse

## Abstract

Uterine leiomyomas usually arise from the uterine body (95%), and rarely from the cervix (0.6%) or other urogenital sites. Lipoleiomyomas are benign, uncommon variants of leiomyomas (0.03–0.2%), histologically composed of smooth muscle cells and mature adipocytes; they usually occur in the uterine body and exceptionally in the cervix. We performed the first systematic literature review of cervical lipoleiomyomas (PRISMA guidelines), presenting five new cases. Including our series, thirty-one detailed cases were reported in the literature (mainly in Asia). The age range was 35–74 years, revealing a higher mean age than conventional cervical leiomyomas (46.5 vs. 39.4 years). Patients were usually multiparous (94%), typically complaining of vaginal bleeding (11/31, 36%), pelvic/abdominal pain (10/31, 32%), and/or urinary disturbances (6/31, 19%) 1 week to 10 months before presentation. Clinical examination revealed a pedunculated tumor (48%), or prolapse of ≥1 pelvic organs (16%). Twenty-four (77%) patients underwent total hysterectomy ± additional surgery; simple myomectomy/excision was performed in five (16%) cases. Only one (3%) of our cases recurred 2 years after partial excision; no evidence of disease was found 13 years after recurrence excision. Adipocytes occupied ≤50% of the tumor volume. Hyaline or myxoid changes and cartilaginous metaplasia were uncommon histological findings. Surgically challenging cases or pregnant patients may require expert gynecologists. Interventional radiology or conservative treatments were rarely proposed.

## 1. Introduction

Uterine leiomyomas are benign smooth muscle tumors, typically found in the uterine body (95% of cases), with a prevalence of 20–40% after 35 years of age [1,2,3,4,5,6,7,8]. Pedunculated submucosal leiomyomas of the uterine corpus may protrude through the cervical os, causing vaginal bleeding and pelvic pain as common symptoms; however, leiomyomas exceptionally arise from the cervix (less than 300 reported cases; frequency: 0.6%) or other urogenital tract locations [1,2,3,4,5,6,7,8]. Different surgical procedures (such as myomectomy or hysterectomy), interventional radiology, or conservative treatments have been proposed to manage uterine leiomyomas; however, surgery of cervical leiomyomas can be more challenging because of the risk of intraoperative hemorrhages or of injuries to adjacent structures, as well as in cases of fertility-sparing approaches or pregnant patients [7,9,10,11].

Lipoleiomyomas (LLMs) are rare histological variants of leiomyomas, being composed of smooth muscle cells mixed with variable extension of mature adipocytes; they account for about 0.03–0.2% of all uterine leiomyomas [1,12,13,14]. Exceptionally, LLMs may arise from the cervix [1,12,13,14], but no systematic literature review (SLR) of cervical LLMs has been previously published.

In the present study, we report a new series of five cervical LLMs; in addition, we have performed the first SLR on these tumors to better delineate their clinicopathologic features, imaging results, treatment, and follow-up.

## 2. Materials and Methods

### 2.1. Our Case Series

Five cases of cervical LLMs were retrieved from our files. The specimens had been fixed in 10% buffered formalin and routinely processed. Formalin-fixed, paraffin-embedded blocks were sectioned, and the resulting slides were stained with hematoxylin and eosin. The following immunohistochemical markers were tested in case 2: smooth muscle actin (clone 1A4, mouse monoclonal, Cell Marque, Rocklin, CA, USA) and CD31 (clone JC70, mouse monoclonal, Cell Marque, Rocklin, CA, USA).

### 2.2. Systematic Literature Review

**Figure 1:**
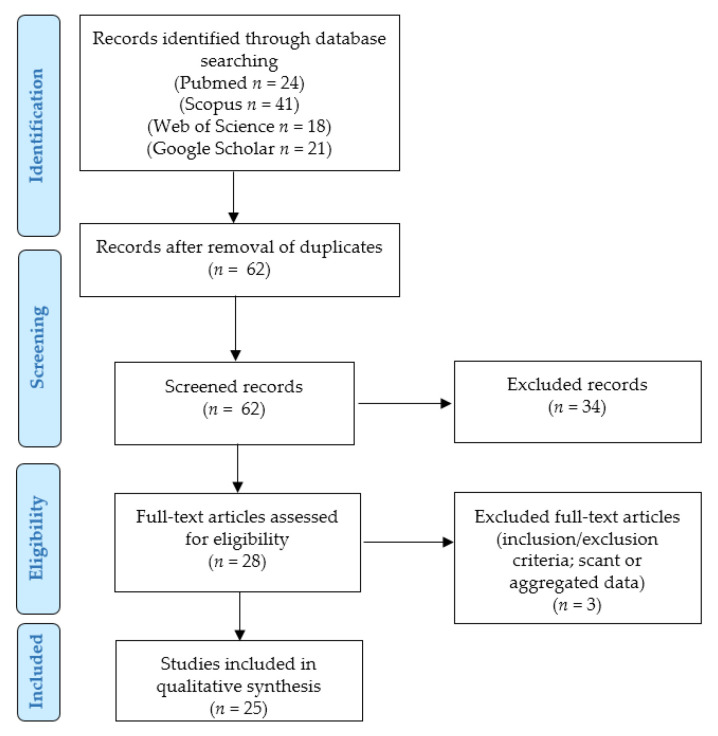
Systematic literature review: PRISMA flow chart.

We performed a SLR according to the “Preferred Reporting Items for Systematic Reviews and Meta-Analyses” (PRISMA) guidelines (http://www.prisma-statement.org/; accessed on 15 October 2022) (Figure 1) to identify the previously reported cases of cervical LLMs.

Our retrospective observational study was conducted through the PICO process:

- Population: human patients with a diagnosis of LLM of the cervix;
- Intervention: any;
- Comparison: none;
- Outcomes: patients’ clinical outcomes (status at last follow-up, and survival and recurrence rates).

We searched for ((lipoleiomyoma OR lipomatous OR lipoleiomyomatous OR (leiomyoma AND (fat OR adipose OR fatty))) AND cervix) in the Pubmed (all fields; 24 results; https://pubmed.ncbi.nlm.nih.gov, accessed on 15 October 2022), Scopus (Title/Abstract/Keywords; 41 results; https://www.scopus.com/home.uri, accessed on 15 October 2022) and Web of Science (Topic/Title; 18 results; https://login.webofknowledge.com, accessed on 15 October 2022) databases. We also searched for “lipoleiomyoma” plus “cervix” or “cervical” in the Google Scholar database (advanced search; words to search in the titles of the articles; 21 results; https://scholar.google.com/schhp?hl=it&as_sdt=0,5; accessed on 15 October 2022). No limitations or additional filters were set. The bibliographic research ended on 15 October 2022. We applied the following:

- Eligibility/inclusion criteria: studies reporting LLMs of the cervix in human patients.
- Exclusion criteria: unclear diagnosis; LLMs of other sites; non-analyzable results (aggregated data).

Two independent authors removed the duplicates and checked the titles and abstracts of all the retrieved results (*n* = 62). After applying the eligibility, inclusion, and exclusion criteria, they selected 28 relevant eligible papers, which were all obtained in full-text format and screened for additional references. After reading the full-texts, three articles were excluded as they were unfit according to the inclusion/exclusion criteria (too aggregated data) [15,16,17]. Two other authors checked the extracted data, and 25 articles were finally included in our study [18,19,20,21,22,23,24,25,26,27,28,29,30,31,32,33,34,35,36,37,38,39,40,41,42]. Data collection was study- and case-related. Continuous variables were analyzed by ranges and mean values, while categorical variables were analyzed as frequencies and percentages.

## 3. Results

### 3.1. Our Case Series

#### 3.1.1. Case 1

**Figure 2:**
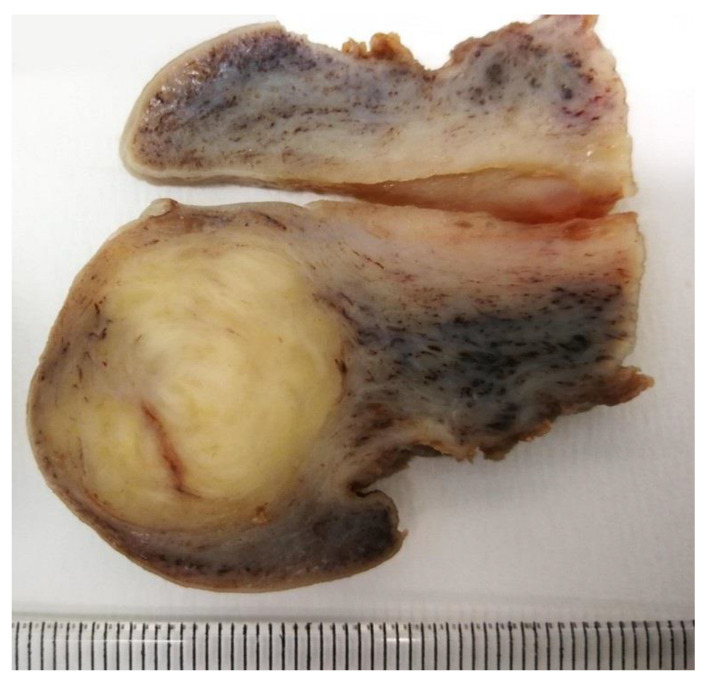
**Case 1: gross section of the uterine cervix.** A 2.1 cm grayish-yellow intramural nodule with mainly regular margins (previously unpublished, original photo).

A 43-year-old woman (P6402) presented with vaginal spotting. The speculum exam revealed a regular cervix with an evident “cystic” lesion on the anterior cervical lip. On transvaginal ultrasounds, the lesion was supposed to be a 2.3-cm cervical leiomyoma; a posterior subserosal leiomyoma of the uterine corpus was also identified. No other relevant findings were reported. The patient was regularly followed up but developed progressive uterine prolapse. Three years later, the patient’s condition worsened, revealing a 3rd-degree uterine prolapse and moderate cysto-rectocele; the woman complained of stress incontinence. A Pap smear resulted in a negative. Total vaginal hysterectomy by morcellement, bilateral salpingectomy, and pelvic floor repair were performed. The uterus was split into three fragments, two of the uterine corpus (10 × 5 × 4 cm and 9 × 5 × 5 cm, respectively) and one of the elongated, hypertrophic cervix (length: 6 cm). On gross examination, a 2.1 cm grayish-yellow intramural nodule with mainly regular margins was identified in the wall of the anterior cervix (Figure 2).

A few other grayish nodules were found in the myometrium of the uterine corpus: the bigger nodule measured 1.6 cm in maximum size. 

**Figure 3:**
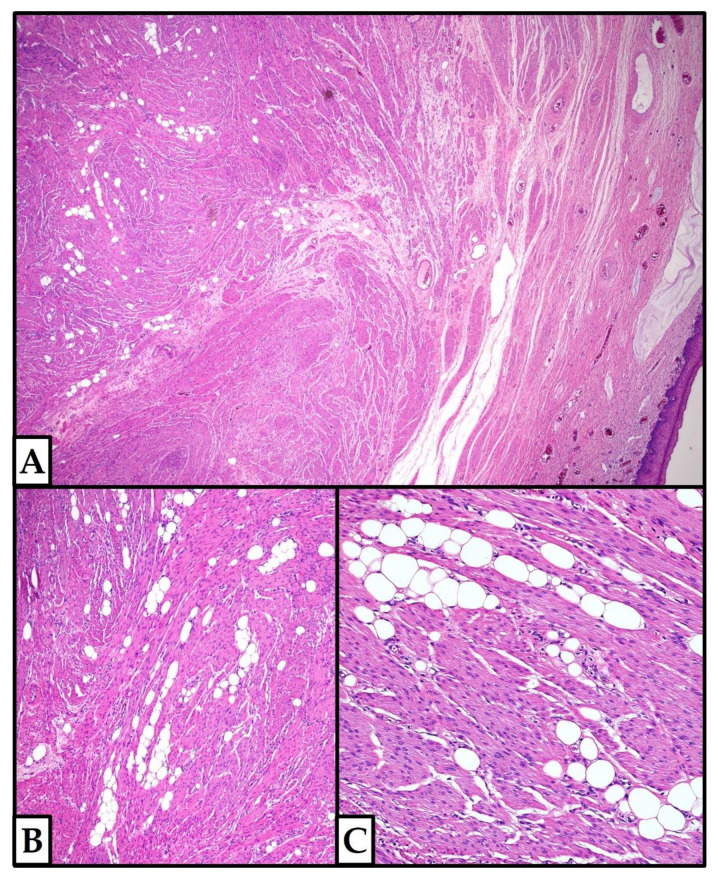
**Case 1: histologic examination.** (**A**) In this area, the tumor nodule (on the right) showed slightly pushing borders; the normal cervical squamous epithelium is present on the left. (**B**,**C**) Details of the cervical lipoleiomyoma: the adipocytes accounted for about 5–10% of the tumor volume. Necrosis, mitoses, and nuclear atypia were absent ((**A**–**C**): hematoxylin and eosin, previously unpublished, original photos; (**A**): 4×; (**B**): 10×; (**C**): 20×).

On histological examination (Figure 3), the cervical nodule was composed of a fascicular proliferation of smooth muscle cells admixed with mature adipocytes (isolated or in small groups); the fatty areas accounted for about 5–10% of the tumor volume.

Necrotic areas, nuclear atypia, mitoses, hemorrhage, regressive changes, vascular proliferation, heterologous components, and vascular invasion were not identified. A diagnosis of cervical LLM was made. The other nodules in the uterine corpus were conventional leiomyomas. A proliferative endometrium and superficial adenomyosis were also reported. The Fallopian tubes were regular, showing a right paratubal serous cystadenoma (diameter: 2.5 cm). No evidence of recurrence was found after 1 year.

#### 3.1.2. Case 2

**Figure 4:**
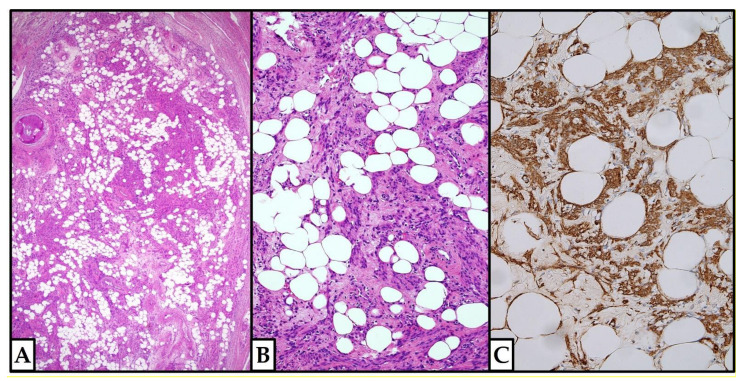
**Case 2: histologic examination.** (**A**,**B**) Well-delimited tumor nodule; the adipocytes accounted for about 45% of the tumor volume. No necrotic areas, mitoses, or nuclear atypia were present. ((**A**,**B**): hematoxylin and eosin; (**A**): 4×; (**B**): 10×). (**C**) Smooth muscle actin immunohistochemical positivity in the smooth muscle cells (10×; clone 1A4, mouse monoclonal, Cell Marque, Rocklin, CA, USA) ((**A**–**C**): previously unpublished, original photos).

A 43-year-old woman (P3003) underwent total abdominal hysterectomy for multiple uterine leiomyomas and adhesiolysis due to three previous caesarean sections and bilateral tubal sterilization (5 years before). The uterus weighed 225 gr and measured 12 × 9 × 8 cm. Two grayish fascicular intramural leiomyomas were found in the uterine corpus, the bigger of which measured 4.2 cm in diameter. A small LLM (cm 0.8) was incidentally found in the uterine cervix; adipocytes accounted for about 45% of the tumor volume (Figure 4). 

No necrotic areas, mitoses, or nuclear atypia were present. On immunohistochemical analysis (Figure 4), smooth muscle actin (clone 1A4, mouse monoclonal, Cell Marque, Rocklin, CA, USA) resulted diffusely positive in the smooth muscle component, while CD31 (clone JC70, mouse monoclonal, Cell Marque, Rocklin, CA, USA) was positive only in the endothelial cells. No evidence of recurrence was found after 6 years.

#### 3.1.3. Case 3

A 42-year-old woman with a 2nd–3rd degree cystocele showed an endometrial polyp and a nodular lesion protruding from the cervix. The nodule was excised, and an endometrial polypectomy was performed. The nodule was well-delimited, grayish, elastic, and fascicular, measuring 3.8 cm in diameter. On histological examination, it revealed a submucosal LLM of the uterine cervix; the adipocytes accounted for about 25% of the tumor volume. No necrosis, nuclear atypia, or mitoses were found. An endometrial polyp was also diagnosed. No evidence of recurrence was found after 17 years.

#### 3.1.4. Case 4

In 2007, a 40-year-old woman without previous significant history underwent hysteroscopy for currently unavailable symptoms; the uterine cavity was regular without mucosal lesions in the endometrium or endocervix, while a hard, sessile, but partially twisted exocervical polypoid lesion of 2 cm in maximum size was identified and partially excised. On histological examination, the polyp was revealed to be a cervical LLM. After 2 years, a pedunculated polypoid gray lesion was found to have grown and protruded from the cervix (maximum size: 5 cm), probably representing a “recurrence” of the previously partially removed LLM. After excision, histological examination confirmed a cervical submucosal LLM. In both specimens, nuclear atypia, necrosis, mitoses, and other worrisome features were absent, and the adipocytic component covered less than 10% of the tumor volume. Thirteen years later, no evidence of disease was found on a gynecological examination. 

#### 3.1.5. Case 5

**Figure 5:**
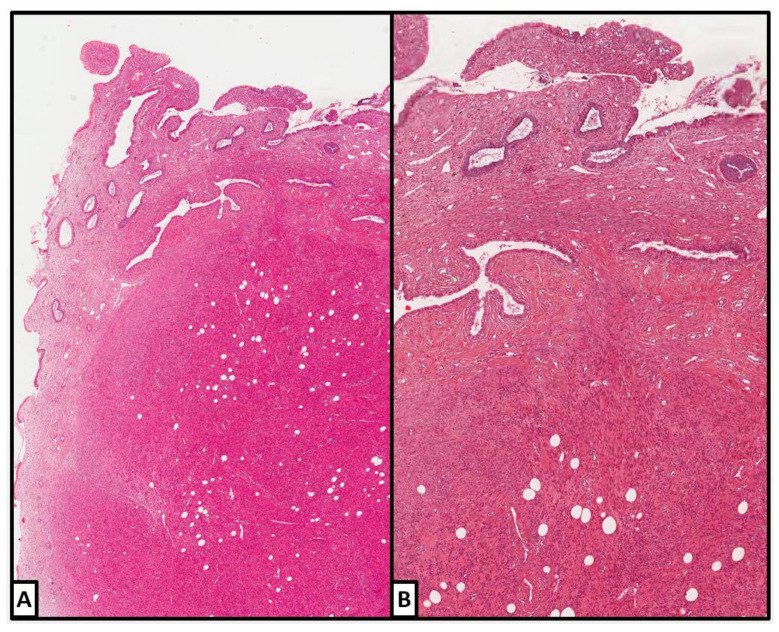
**Case 5: histologic examination.** (**A**,**B**) Well-delimited tumor nodule growing under the cervical mucosa; the adipocytes accounted for about 5% of the tumor volume. Necrosis, mitoses, or nuclear atypia were absent. ((**A**,**B**): hematoxylin and eosin; (**A**): 4×; (**B**): 10×; previously unpublished, original photos).

In 2010, a 44-year-old woman underwent total hysterectomy and bilateral salpingo-oophorectomy for a right ovarian cystic multilocular Sertoli cell tumor of 8 cm in maximum size (pTNM stage: pT1a; FIGO stage: IA). A 1-cm polypoid endocervical nodule was also identified; on histological examination, it was revealed to be a cervical LLM without necrosis, nuclear atypia, or mitoses. The adipocytes accounted for about 5% of the tumor volume (Figure 5); after surgery, the patient was lost at follow-up.

### 3.2. Systematic Literature Review Results

#### 3.2.1. Overview

Globally, including our series, only 44 cases of cervical LLMs were found in the literature [15,16,17,18,19,20,21,22,23,24,25,26,27,28,29,30,31,32,33,34,35,36,37,38,39,40,41,42]. The majority of cases were diagnosed in Asia (27/44, 61%), followed by North America (9/44, 20%), Europe (6/44, 14%), and Africa (2/44, 5%), while no cases were reported in other continents. The majority of cases were described in India (13/44, 30%) [19,20,21,22,25,27,30,31,32,33,34,36,39], the United States (9/44, 21%) [16,28,38], and Turkey (8/44, 18%) [15,17,18,23]. According to our review, cervical LLMs were not described in North America, except for the United States, while the two African cases were reported in North Africa (one in Egypt and one in Morocco) [29,35]. For our review, all the European cases were described in Italy (6/44, 14%), including our series (the largest Italian and European one) and the first-reported cervical LLM [41]. Other cases were found in Japan (4/44, 9%) [26,37,40], South Korea (one case) [42], and China (one case) [24].

As reported in the Materials and Methods, we had to exclude the following three series from further analysis because the clinicopathologic features were not reported in detail for each case:

1. Akbulut et al. described 76 LLMs (2.9% of the uterine LLMs in their files) arising in the uterine corpus (69/76, 90.7%), cervix (5/76, 6.5%), retroperitoneum (one case), and broad ligament (one case) [15].
2. Bolat et al. found 10 (1.4%) LLMs among 707 uterine leiomyomas; only 1/10 (10%) of these cases arose in the cervix [17].
3. Wang et al. reported a series of 50 LLMs occurring in the cervix (seven cases, 14%), uterine corpus (43 cases, 86%), retroperitoneum (one case), and broad ligament (one case) [16].

So, including our cases, only 31 detailed cases of cervical LLMs were reported in the literature, according to our systematic literature review (Table 1) [18,19,20,21,22,23,24,25,26,27,28,29,30,31,32,33,34,35,36,37,38,39,40,41,42]. 

The age range of the reported 31 patients was 35–74 years (mean: 46.5 years; median: 43 years). Ten women (32%) were clearly or probably postmenopausal [20,22,23,28,31,37,40,41,42], while 21/29 (68%) cases were pre- or peri-menopausal [18,19,21,24,25,26,27,29,30,32,33,34,35,36,38,39]. Information about parity was available in only 16 (52%) cases [18,19,21,25,28,29,31,32,33,35,36,38,41,42]; 15/16 (94%) women were multiparous [18,19,21,25,28,29,31,32,33,35,36,41,42], while only 1 (6%) of 16 patients had only one child [38]; no woman was clearly nulliparous.

#### 3.2.2. Clinical Symptoms and Signs

The most frequent symptoms were vaginal bleeding (11/31, 36%) [19,20,21,26,29,30,35,36,39,41] and pelvic/abdominal pain (10/31, 32%) [19,20,21,23,29,30,31,32,33,37], followed by urinary disturbances (6/31, 19%) (1 not otherwise specified [22]; 2 stress incontinence [25]; 1 difficulty in micturition [30]; 2 hesitancy/difficulties in passing urine [19,34]). Occasionally, patients experienced low abdominal distension (three cases, including one due to a concomitant ovarian tumor) [37,38,40], vaginal discomfort (one case) [32], inguinal pain (one case) [18], or dyspepsia (one case) [33] at presentation.

The time interval between the onset of symptoms and the clinical exam ranged from 1 week to 10 months. Thirteen patients (42%) complained of one symptom [18,22,23,25,26,31,34,35,36,37,39,40,41], six women (19%) experienced two symptoms [20,21,29,32,33], and two patients (6%) presented three symptoms [19,30]. In nine (29%) cases, data about clinical symptoms were unavailable [24,27,28,38,40], while one (3%) patient was clearly asymptomatic [42].

Fifteen patients (48%) showed a mass/polyp that was described as protruding through the vagina, pedunculated, and/or increased in size [18,19,21,22,25,28,30,33,34,36,40,42]. 

Globally, 5/31 (16%) patients experienced a prolapse of pelvic organs (PPO) [22,28,40]. In detail, two patients (6%) revealed a uterine prolapse (3rd grade in our first case; unknown degree in the other patient) [40]; the ages of these women were 43 and 74 years, respectively. Moreover, the cervix was described as elongated in two additional premenopausal patients (35 and 39 years of age, respectively) [25,32] and distorted in another 35-year-old woman [29], while a 58-year-old lady experienced a 3rd-degree vaginal prolapse [22] (total of women with utero-vaginal abnormalities: six cases, 19%). Three patients (10%) presented with cystocele [40], which was associated with uterine prolapse ± rectocele in 2/3 (67%) cases. Two women (6%) presented with rectocele [28], being associated with uterine prolapse and cystocele in our first case. 

Other findings included synchronous leiomyomas of the uterine body (*n* = 10, 32%) [22,23,25,27,28,30,34,40], left ovarian mucinous cystadenoma (*n* = 1, 3%) [40], right paratubal serous cystadenoma (*n* = 1, 3%), 2.1-cm adult granulosa cell tumor of the right ovary (*n* = 1, 3%) [38], 8-cm Sertoli cell tumor of the right ovary (pT1a) [42], and cervical H-SIL (*n* = 1, 3%) [40]. Pap smears were performed in three other cases (10%) (including our first case), always resulting in a negative result [19,42].

#### 3.2.3. Imaging

On ultrasound examination, the tumors were described as heterogeneous/complex echoic (three cases, 10%) [31,33,38], heterogeneous hyperechoic with minimal vascularity (one case, 3%) [18], heterogeneous hypoechoic (one case, 3%) [21], hypoechoic (one case, 3%) [36], isoechoic (one case, 3%) [32], or hyperechoic (four cases, 13%) [29,30,35,42]. Doppler imaging revealed high resistance blood flow in two cases (6%) [19,29], while a tumor was not vascularized (3%) [42]. Walid et al. identified fibroid and fatty areas on ultrasound examination [38].

Computed tomography scans revealed intralesional fat in two cases (6%) [20,42]. The case of Sharma et al. was described as hypodense with small enhancing solid areas [30], while another LLM was lobulated, heterogeneously hypodense, with an enhanced solid component and multiple septa of variable thicknesses [33]. Finally, Kim et al. [42] described a hypodense, well-delimited tumor without cystic areas or calcifications.

Magnetic resonance imaging information was available for three patients (10%): in two cases (6%), these LLMs were hyperintense on T1/T2 scans and heterogeneously hypointense on fat-suppressed T2 scans [20,23], while the third case was heterogeneous on T2 scans with intense contrast-enhancement [18].

#### 3.2.4. Treatment and Follow-Up

Twenty-four (77%) patients underwent total hysterectomy (15 abdominal, 4 vaginal, 4 simple, and 1 not otherwise specified) [18,19,21,22,23,25,26,27,30,31,32,33,34,35,36,37,38,39,40,41]; morcellement was performed in two (6%) cases [19]. Additional surgeries included unilateral (three cases) [21,32,36] or bilateral (11 cases) salpingo-oophorectomy [23,27,30,31,33,35,37,38,40,41], bilateral salpingectomy (two cases) [18,41], bilateral salpingectomy + unilateral oophorectomy (one case) [19], pelvic floor repair (three cases) [22,25], omentectomy (one case) [33], and internal hernia repair (one case) [27]. Four (13%) patients had previously undergone previous uni- or bi-lateral salpingectomy [27,32,36].

Simple myomectomy or excision of the nodule protruding through the vagina were performed in five (16%) cases [28,29,42]; 1/5 (20%) patients underwent additional bilateral salpingectomy [42]. In one (20%) of the five cases, the excision was partial, and the tumor recurred after 2 years; the recurrence was excised. Finally, information concerning treatment was unavailable for two women [20,24].

Follow-up information was reported in 16/31 cases (52%), despite frequently being incomplete: post-operative complications were not reported in any case [21,26,27,29,30,31,35,37,40,41,42]. Only one (3%) of 31 LLMs recurred 2 years after partial excision; no evidence of disease was found on a gynecological exam 15 years later. No tumor recurrence was found from 6 weeks to 17 years after surgery in the remaining cases (mean 38 months) [21,26,27,29,30,31,35,37,40,41,42]. Data about pregnancies after surgery were unavailable.

#### 3.2.5. Gross Findings

When data were available, the LLMs were located in the anterior (5/31, 16%) [19,20,28,29], posterior (8/31, 26%) [21,22,25,30,32,33,35,41], or right lateral (3/31, 10%) [23,27,40] cervix, while the tumor occupied the whole cervix in 2/31 (6%) cases [34,36].

Four LLMs (13%) were located near to the isthmus, including a tumor also extending to the parametrial tissues [22,26,35,38]. Five additional cases (16%) were found deeply in the cervix, being reported as “subserosal” [22,26,31,33,40]. The peritoneum of the uterine serosa usually reflects at the isthmus or slightly below on the posterior cervix, while the anterior cervix is less peritonealized, as the serosal reflection occurs higher on the anterior part; so, if the term “subserosal” was correctly used, implying at least a partial covering of the tumors by the uterine serosa, these LLMs may have been located near to the isthmus or in the posterior part; indeed, 2/5 (40%) tumors were also qualified as posteriorly localized. In addition, four (13%) cases were intramural [23,27,30,40] and two (6%) of our LLMs were described as submucosal.

On gross examination, the nodules were solid, usually well-circumscribed [20,21,22,23,29,30,31,33,38,40,41,42], and occasionally multilobulated [31]. A whorled/swirled cut surface was confirmed in five cases [27,39,40,41]. A few cystic areas were identified in one case [34]. The consistency was described as soft [25,38,42], elastic hard [40], firm [18,28,34], or soft to firm [33,36]. In one case, the tumor was hard but friable and vascularized [19]. The color of the nodules was usually gray/white with yellowish areas [21,22,23,25,26,27,30,31,33,36,37,40]; a LLM was fleshy [38], while brownish areas were present in two cases [33,36]. Conversely, at least in five cases, yellow areas seemed not to be identified on macroscopic examination (color: whitish [34]; pale-gray [19,41]; pale white [42]). The tumor size range was 0.8–20 cm (mean: 7.4 cm) [18,19,20,21,22,23,24,25,26,27,28,29,30,31,32,33,35,36,37,38,39,40,41,42]. 

#### 3.2.6. Histopathological Features

When data were available, adipocytes occupied ≤50% of the tumor volume in the cases analyzed in our review. Three studies were excluded from our analysis as they reported data on cervical LLMs aggregated with those of LLMs arising in the uterine body or extrauterine sites [15,16,17]. In the series of Wang et al. (*n* = 50; including seven cervical cases), the distribution of adipocytes widely varied, showing a mean value of 36% [16]. In the study of Bolat et al. (*n* = 10; including one cervical case), adipocytes occupied 5% to 95% of the tumors [17]. Finally, Akbulut et al. (*n* = 76; including five cervical cases) classified the LLMs according to the extension of intratumor adipocytes as grade 1 (minimal, focal) (*n* = 35, 46%), grade 2 (moderate) (*n* = 3, 3.9%), or grade 3 tumors (abundant, multifocal, and evenly distributed) (*n* = 38, 50%) [15]. One cervical LLM showed extensive hyaline degeneration, as the smooth muscle component was replaced by diffuse extracellular collagenous deposits in some areas with minimal alteration in the adipocytic topography [31]. Another case revealed areas of myxoid change and several foci of mature cartilage with haphazardly-distribute islands of mature fat [41].

Nuclear atypia, mitotic figures, and necrotic areas were absent in all the cases, including our recurrent LLM. Thick-walled blood vessels were occasionally described, without vascular proliferation.

Limited information is available about the immunohistochemical profile of cervical LLMs, as immunohistochemistry was performed in only 11/31 (35%) cases [19,21,23,26,27,30,37,40,41] and each marker has only been occasionally tested (Table 2).

The proliferation (Ki-67/Mib-1) index was ≤1% in both the smooth muscle and adipocytic components of the three tested cases [19,26,37]. 

Molecular analysis was not performed on cervical LLMs. 

## 4. Discussion

To the best of our knowledge, we are here presenting the largest Italian and European series of cervical LLMs and the first SLR on this topic.

The histogenesis of LLMs is still unknown, including the following hypotheses: lipoblastic differentiation of misplaced embryonic progenitor cells; metaplastic changes of connective or smooth muscle tissue into adipocytes; perivascular adipocytes; traumatic displacement of fat tissue; pluripotent cell migration along the uterine nerves and vessels and fatty infiltration [13,15]. 

In the normal cervical stroma, smooth muscle fibers—from which leiomyomas or LLMs can arise—are more typical in the endocervix than in the exocervix. It is also controversial if mature adipocytes are normal or heterotopic constituents of the cervical stroma in women of various ages (Doldan et al.: 15–57 years) [43,44]. Being more frequent in the deep stroma (also non-contiguous to the parametrial fat), adipocytes may also be found in the superficial cervix, and detected by cervical biopsies or conizations. Normal fat distribution is haphazard, not forming a circumscribed tumor (such as in LLMs or lipomas) [43,44]. 

Pure malignant or benign lipomatous tumors (liposarcomas or lipomas) or other rare mesenchymal tumors with occasional foci of adipocytic differentiation (such as solitary fibrous tumors) also enter the differential diagnosis with LLMs; they are usually reported in the uterine body, exceptionally occurring in the cervix [14,45,46,47,48,49,50,51,52]. Lipomas are entirely composed of fat tissue, while liposarcomas show malignant histological features that were absent in our series. Solitary fibrous tumors have a haphazard arrangement of spindled to ovoid cells arranged around branching and dilated vasculature within a variably collagenous stroma; desmin is expressed by a subset of cases while STAT6 positivity is typical [14,45,46,47,48,49,50,51,52].

Clinical guidelines are increasingly recommending that gynecological cancers should be treated in specialized centers by expert gynecologic surgeons and oncologists; however, the surgical treatment of benign tumors can also be challenging [7,53,54,55,56]. Cervical leiomyomas (CLs) represent an independent factor influencing the operation time in minimally invasive surgery [53,54]. Being close and often adherent to the bladder, rectum, and/or ureters, their surgical treatment can be difficult, especially in the case of difficult cleavage [57,58,59]. Large tumor size, close relation to uterine vessels, and increased tumor neovascularization are risk factors for bleeding; indeed, large tumors can shift pelvic organs, nerves, and vessels, reducing the surgical accesses and making the suture repair challenging, with a consequently increased risk of surgical injuries and major bleeding [57,58,59,60,61].

Some authors suggested that preoperative gonadotropin-releasing hormone (GnRH) agonists (23% of CLs), the tourniquet method, intraoperative injection of vasopressin-epinephrine into the myometrium (72% of CLs), and permanent (POUA) or temporary (TOUA) occlusion of the uterine artery (36 bilateral uterine arteries ligation; 1 temporary blocking of the uterine artery blood flow with vessel clips; 1 preventive hypogastric artery ligation; 36 bi- or uni-lateral internal iliac artery balloon occlusion catheters) could reduce the risk of bleeding during CL-myomectomy [7]; these factors may also decrease the risk of recurrence [7,60,61,62,63]. However, ligation of the uterine artery can be challenging if large CLs limit the access to the retroperitoneal pelvic space [64,65]. TOUA may have a less negative impact on uterine and ovarian function than POUA [64,65]. Moreover, some patients’ conditions (such as pregnancy and coagulopathies) may increase the difficulties of the surgical procedures and favor bleeding. Pregnancy (especially in the late stages) increases the uterine blood flow and the size of, at least, a subset of leiomyomas, which can be further expanded by the effects of pregnancy hormones [66,67,68,69,70,71,72].

A recent systematic literature review [7] focused on conventional CLs (*n* = 214), excluding the LLMs analyzed by our study due to a different research method; treatment approaches included surgery (187/214, 87%), interventional radiology treatment (IRT) (20/214, 9%), surgery + IRT (1/214, 1%), and exclusive conservative management (6/214, 3%; all pregnant patients). Surgical procedures comprised myomectomy (*n* = 127, 67.5%), hysterectomy (*n* = 54, 28.7%), and trachelectomy (*n* = 7, 3.7%). Only 7/124 (5.6%) patients with available data experienced post-operative complications after laparoscopic myomectomy (1 paralytic ileus + surgical bed abscess; 1 retroperitoneal hematoma; 1 postoperative fever) [58,73,74], laparotomic myomectomy during a cesarean section (1 intraoperative hemorrhage requiring hysterectomy) [75], laparoscopic-assisted vaginal hysterectomy (1 intraoperative hemorrhage; 1 postoperative infection) [65], or vaginal hysterectomy (1 urinary tract infection) [76]. In our review, total hysterectomy (± additional surgical procedures), simple myomectomy/excision, and unclear treatment were performed in 77%, 16%, and 7% of cervical LLMs, respectively; surgical complications were not reported in any cases, also in tumors of greater size (range 0.8–20 cm; mean: 7.4 cm) [18,19,20,21,22,23,24,25,26,27,28,29,30,31,32,33,34,35,36,37,38,39,40,41,42].

The mean age of our patients with cervical LLMs was slightly higher, compared to the CLs of Ferrari et al. (46.5 vs. 39.4 years); we probably included more postmenopausal women (10/31, 32% vs. 5/57, 9%), but the fertility status was available for only 57/214 (27%) patients in the review of Ferrari et al. [7,20,22,23,28,31,37,40,41].

The prevalence of uterine leiomyomas in pregnant women ranges approximately from 3% to 10%, but CLs are even rarer [7]. CLs can be associated with infertility, pregnancy loss, obstructed labor, an increased chance of malpresentation (25% in a series of 17 cases) [75], dystocia, hemorrhages, infections, tumor degeneration/regression changes (infarction, abscess, or cystic formation), and/or the need for hysterectomy [7,75,77,78]. 

Ferrari et al. identified only 23 (40%) pregnant patients and one (2%) puerperal patient with CL; 20/23 (87%) women were treated conservatively during their pregnancy, including patients who subsequently underwent surgery (14/20, 70%) or were still conservatively treated (6/20, 30%) after delivery [7]. Two (33%) women of the last group developed complications requiring hysterectomy, including: (1) persistent fever unresponsive to medical treatment (infection, tumor necrosis, endometritis, and smooth muscle inflammation); (2) uterine atony + hemorrhage [75]. Moreover, a CL spontaneously prolapsed out of the vagina 4 days after an emergency cesarean section for obstructed labor at 37 weeks; the woman had been conservatively treated during pregnancy. After a rubber ring pessary was placed, the tumor decreased in volume without symptoms after 6 weeks [79].

Only three (13%) patients with CL were treated with surgery (2 vaginal myomectomy at 15 and 36 weeks of gestation, respectively) or IRT (1 super-selective uterine fibroid embolization) during pregnancy [7,64,80,81]. No relevant complications were reported in these cases, but prolapse and/or premature rupture of membranes with chorioamnionitis and fetal loss could sometimes happen [64,75,78,82,83]. Vaginal myomectomy may be feasible in selected patients during pregnancy or delivery, despite the risk of increased hemorrhage or other complications [7,64,75]. Pedunculated, easily accessible CLs can also be more easily removed with fewer consequences in pregnant patients compared to intraparietal cervical or isthmic leiomyomas; large CLs seemed to be correlated with adverse outcome [75]. Due to the risk of labor obstruction, most of the patients who were followed up or treated during their pregnancy underwent a cesarean section, but vaginal delivery was occasionally carried out [64,75]; limited data were available for reliable comparisons. 

Despite the still limited results, IRT can be promising for the treatment of CLs, especially in cases of surgical contraindications or the patient’s desire for uterine preservation. These women may benefit from super-selective embolization of the leiomyoma or cervicovaginal artery; however, subsequent pregnancies after uterine embolization have a statistically significantly higher rate of spontaneous abortion (56% vs. 10.5%), malpresentation (20%), and cesarean section (80%) compared to those following surgical uterine artery occlusion [84]. In the study of Ferrari et al., IRT procedures were successful in 10/18 (55.5%) cases and in the two cases undergoing super-selective cervico-vaginal artery embolization and UFE (pregnant patient), respectively [7,80]. The largest treated tumor was of 9 cm in maximum size [7]. Our cases were not treated according to these procedures; further studies are required. Finally, information about the exclusive medical treatment of CLs or cervical LLMs is scant [7,64,70,73,85].

Long-term follow-up was rarely available for CLs or LLMs [7,21,26,27,29,30,31,35,37,40,41,42]. Limited data are available about pregnancies after surgery for CLs or cervical LLMs; our patients were not pregnant at the time of diagnosis, and details about subsequent pregnancies were unavailable [7]. Only one (3%) of our LLMs and one (2%) of the 45 CLs analyzed by Ferrari et al. recurred 2 years after partial excision and 1 year after radical abdominal trachelectomy, respectively; in the latter case, an abdominal hysterectomy was carried out, while our patient underwent excision of the recurrent tumor, becoming free of disease 13 years later [7,54].

Morcellement may favor tumor recurrence; iatrogenic parasitic leiomyomas (IPLs) may result from morcellated leiomyomas that have implanted far from the uterus, developing an independent blood supply from adjacent structures. The overall incidence of IPLs after laparoscopic surgery using a morcellation approach accounts for 0.12–0.95% (0.20–1.25% after laparoscopic myomectomy). The time interval between the initial surgery and diagnosis ranges from 3 to 8 years [86,87]. Only two (6%) of our LLMs were morcellated [19]; none of them recurred or developed IPLs.

Malignant transformation of leiomyomas/lipoleiomyomas is exceptional; morcellation of an unsuspected leiomyosarcoma might worsen the prognosis [88,89,90]. The risk of finding a sarcoma in morcellated surgical specimens ranges from 1:770 to <1:10,000; so, surgeons frequently use a laparotomic approach, which may imply increased morbidity, if compared to minimally invasive surgery [86,87,91,92]. None of our cases had undergone malignant transformation. An accurate preoperative evaluation of cervical LLMs should be performed, and the risks and benefits of the different surgical approaches should be discussed with patients [91]. 

In our series, 5/31 (16%) patients experienced a PPO, showing a variable association of uterine prolapse (UP) (*n* = 2; 6%), vaginal prolapse (*n* = 1; 3%), cystocele (*n* = 3; 10%), and/or rectocele (*n* = 2; 6%) [22,28,40]. In our series, the mean age of patients with PPO and cervical LLM was 53.4 years. Indeed, about half of women after age 50 years usually show PPO; UP accounts for 50% of all PPO cases, but its incidence is frequently underestimated as early UPs are often asymptomatic [93,94,95,96]. Bulging tumors, pressure sensation in the pelvis or vagina, and/or lower back pain are the most common complaints of patients with UP, followed by urinary symptoms (such as urgency, urinary frequency, and incomplete emptying of the bladder) and dyspareunia. These symptoms gradually worsen as the prolapse increases, but their severity is usually unrelated to the UP degree; they can also be due to (or increased by) CLs [97,98]. Globally, women have an 11–19% lifetime risk of being treated for PPO, but this risk is expected to rise to 45% in the future due to the increasing population age [96,97,98]. Well-known risk factors are childbirth, age, obesity, chronic constipation, and connective disorders [99,100]. Prolonged labor (>12 h) and operative vaginal delivery with the use of forceps have been reported in most women with UP [101]. 

Pedunculated submucosal uterine leiomyomas prolapsing through the cervical canal can mimic a UP, although some studies suggest their potential role in causing UP/PPO [76,102,103]. Prolapse of leiomyomas occurs in ~2.5% of women undergoing surgery for leiomyoma [104]; its causes have not been completely clarified. Prolapsing submucosal leiomyomas of the uterine body, as well as intraparietal or pedunculated CLs, especially if large and heavy, can drag down the cervix, urinary bladder, and/or vaginal wall, predisposing to PPO and cervical elongation. While the vaginal part of the cervix may prolapse with the vagina, the supravaginal portion (well supported by Mackenrodt’s ligament) can be stretched and elongated due to the presence of a CL [76,103].

In younger women of childbearing age, hypertrophic cervical elongation can mimic UP because it has a similar presentation, but it lacks other anatomical defects (such as descent of the uterus and level 1 ligament impairment) [105]. Although the pathophysiology still remains unknown, prolonged labor and cervical dystocia seem to be associated with this condition [106]. In our series, the cervix was described as elongated or distorted in three premenopausal patients under 40 years of age [25,29,32]. CLs may mimic congenital cervical elongation; however, in this condition, the fornices are deep and the elongation is limited to the vaginal portion [76,103]. 

## 5. Conclusions

Compared to leiomyomas of the uterine body, CLs were rarely described in literature; LLMs represent a rare variant of uterine leiomyomas, and only 31 cases were found to arise from the cervix. Vaginal bleeding, pelvic pain, urinary disturbances, and a tumor protruding through the cervical os are the main complaints; 5/31 (16%) patients were associated with a PPO, while 3/31 (10%) cases showed an elongated/distorted cervix, potentially playing a role in their pathogenesis by dragging down the pelvic organs (as to the tumor size and weight). Myomectomy or hysterectomy is the therapy of choice based on the desire for pregnancy and anatomic features; however, surgery may be challenging, requiring expert gynecologists. Interventional radiology or conservative treatments have rarely been proposed to manage CLs; further studies are required.

## Figures and Tables

**Table 1:** Lipoleiomyomas of the uterine cervix.

Authors	Age	Size (cm)	Clinical Features	Site	Therapy	%Fat	Follow-Up
Palicelli et al., 2022: case 1	43	2.1	P6402, VB, uterine prolapse (progressing to 3rd degree), moderate cysto-rectocele, UD	A	TVH (mor) + BSO + PFR	5–10%	NED, 12 mo
Palicelli et al., 2022: case 2	43	0.8	P3003, BS (sterilization, 5 years before)	NR	TAH + adhesiolysis	45%	NED, 6 years
Palicelli et al., 2022: case 3	42	3.8	Pr, cystocele (2nd-3rd degree)	Sm	EXC	25%	NED, 17 years
Palicelli et al., 2022: case 4	40	2; 5 (REC)	Pr/Pe	Sm	Partial EXC	<10%	REC, 2 years; NED, 13 years after REC
Palicelli et al., 2022: case 5	44	1	Pr/Pe. Concomitant right ovarian Sertoli cell tumor	NR	TH + BSO	5%	NR
Kim et al., 2022 [42]	55	5.8	G2P2, asymptomatic, normal BMI (23.3), suddenly increasing cervical mass after 4 years of follow-up	NR	Pelviscopic EXC + BS	NR (significant component, 20%?)	NED, 3 years
Mihmanli et al., 2020 [18]	39	8.3	G2P2, inguinal pain (increased during sexual intercourse), Pr	NR	TH + BS	NR	NR
Agrawal et al., 2020 [19]	45	13	G2P2, VB, PP, Pr, UD (6 mo), treated hypothyroidism (5 years)	A	TAH (mor) + LSO + RS	NR	NR
Ravikanth et al., 2020 [20]	68	4.3	VB, PP (2 we)	A	NR	NR	NR
Rathore et al., 2018 [21]	37	13	G2P2L2A1, VB (3 mo), PP (1 mo), Pr (1 we)	P	TAH + USO	<50% (#)	NED, 3 mo
Bannur et al., 2017 [22]	58	6	UD (22 we), Pr (6 mo), 3rd degree vaginal prolapse	P, Is, Su	VH + PFR	NR	NR
Şengiz Erhan et al., 2017 [23]	53	2	PP (3 mo)	R, In	TAH + BSO	NR	NR
Ye et al., 2016: case 3 [24]	44	6.5	NR	NR	NR	35%	NR
Adaikkalam et al., 2016 [25]	39	8	Multiparous, UD (1 we), Pr (3 mo)	P	VH + PFR	NR	NR
Terada T., 2015: case 1 [26]	47	10	VB	Is, Su	SH	50%	NED, 5 mo
Singh et al., 2015 [27]	37	9	Tubal sterilization	R, In	TAH + BSO + HR	NR	NED, 6 we
Barnard et al., 2015 [28]	50	4	Multiparous, LR-IUD (5 mo), Pr (3 mo), rectocele	A	EXC	NR	NR
El-Agwany AS, 2015 [29]	35	5	G2P2, VB, PP (3 mo)	A	SM	NR	NED
Sharma et al., 2015 [30]	39	3	VB, PP, Pr, UD (4 mo)	P, In	TAH + BSO	NR (°)	NED
Mandal et al., 2015 [31]	48	19	P2 + 0, PP (4 mo)	Su	TAH + BSO	<50% (#) ($)	NED, 6 mo
Kalyankar et al., 2014 [32]	35	15	P3L3, salpingectomy (12 years before), PP, VD (4 mo)	P	TAH + RSO	NR	NR
Goyal et al., 2014 [33]	40	20	G3P3, PP, Dysp (45 days), Pe	P, Su	TAH + BSO + Om	NR	NR
Chaudhari et al., 2014 [34]	40	NR	UD (2 mo), Pr (6 mo)	WC (*)	TAH	NR	NR
Fagouri et al., 2014 [35]	39	6	G4P4, VB (3 mo)	P, Is	TAH + BSO	NR	NED
Agrawal et al., 2014 [36]	38	5	P3G3A0, salpingectomy (15 years before), VB (3.5 mo), Pr	WC	TAH + RSO	NR	NR
Terada T., 2011 [37]	73	18	PP, lower abdominal mass	NR	SH + BSO	40%	NED, 36 mo
Walid et al., 2010 [38]	48	5.5	G1P1, slowly growing cervical mass	Is, Par	TAH + BSO	NR	NR
Mohan et al., 2002: case 3 [39]	38	2.5	VB	NR	TAH	focal	NR
Shintaku M, 1996: case 4 [40]	74	3.5	Uterine prolapse, cystocele	In	VH	<50% (#)	NED
Shintaku M, 1996: case 5 [40]	60	4.2	Low abdominal distension (due to ovarian tumor), Pe	R, Su	SH + BSO	<50% (#)	NED
Volpe et al., 1992 [41]	51	15	Multiparous, VB (10 mo)	P	TAH + BSO	<50% (#) (@)	NED, 5 mo

(*) except for a spared small right anterior area; (#): smooth muscle cells predominated over the interspersed adipocytes (small groups or single cells). One case [41] also revealed myxoid areas and several islands of mature cartilage. (°): quite diffuse as to the photos. ($): the myogenic component was often replaced by diffuse extracellular collagenous deposits, with minimal alteration in the adipocytic topography (lipoleiomyoma with extensive hyaline degeneration). (@): areas of myxoid change, haphazardly arranged islands of mature fat, and several islands of mature cartilage. A: anterior; BS: bilateral salpingectomy; BSO: bilateral salpingo-oophorectomy; Dysp: dyspepsia; EXC: Excision of the tumor (conservative surgery); HR: incisional hernia repair; In: intramural; Is: towards the isthmus; LR-IUD: levonorgestrel-releasing intrauterine device; LSO: left salpingo-oophorectomy; mo: months; mor: morcellement; NED: no evidence of disease; NR: not reported; Om: omentectomy; P: posterior; Par: parametrial; Pe: peduncolated; PFR: pelvic floor repair; PP: pelvic/abdominal pain; Pr: protruding vaginal mass; R: right lateral; REC: recurrence; RS: right salpingectomy; RSO: right salpingo-oophorectomy; SD: stable disease; SH: simple hysterectomy; Sm: submucosal; SM: simple myomectomy; Su: subserosal; TAH: total abdominal hysterectomy; TH: total hysterectomy; TVH: total vaginal hysterectomy; UD: urinary disturbances; USO: unilateral salpingo-oophorectomy; VB: vaginal bleeding; we: weeks; VD: vaginal discomfort; VH: vaginal hysterectomy; WC: whole cervix.

**Table 2:** Lipoleiomyomas of the uterine cervix: immunohistochemical profile.

Marker	Smooth Muscle Cells	Adipocytes
Vimentin [26,30,37,41]	100% (4/4)	100% (3/3)
Desmin [19,23,26,27,30,37,40,41]	89% (8/9)	0% (0/2)
Smooth muscle actin [19,23,26,27,30,37,40]	100% (10/10)	0% (0/2)
h-caldesmon [19]	100% (1/1)	
Estrogen receptor [26,30]	100% (1/1)	50% (1/2)
Progesterone receptor [26,30]	100% (1/1)	50% (1/2)
pan-cytokeratins (AE1/3 and CAM5.2) [26,37]	0% (0/2)	0% (0/2)
S-100 [19,23,26,37,40,41]	0% (0/2)	100% (7/7)
HMB-45 [26,37]	0% (0/2)	0% (0/2)
p53 [26,37]	0% (0/2)	0% (0/2)
MDM2 [26,37]	0% (0/2)	0% (0/2)
CDK4 [26,37]	0% (0/2)	0% (0/2)
CD34 [37]	0% (0/1)	0% (0/2)

## References

[1] WHO Classification of Tumours Editorial Board (2020). Female Genital Tumours: WHO Classification of Tumours.

[2] Solomon L.A., Schimp V.L., Ali-Fehmi R., Diamond M.P., Munkarah A.R. (2005). Clinical update of smooth muscle tumors of the uterus. J. Minim. Invasive Gynecol..

[3] Laganà A.S., Alonso Pacheco L., Tinelli A., Haimovich S., Carugno J., Ghezzi F., Mazzon I., Bettocchi S. (2019). Management of Asymptomatic Submucous Myomas in Women of Reproductive Age: A Consensus Statement from the Global Congress on Hysteroscopy Scientific Committee. J. Minim. Invasive Gynecol..

[4] Vitale S.G., Sapia F., Rapisarda A.M.C., Valenti G., Santangelo F., Rossetti D., Chiofalo B., Sarpietro G., La Rosa V.L., Triolo O. (2017). Hysteroscopic morcellation of submucous myomas: A systematic review. Biomed. Res. Int..

[5] Tiltman A.J. (1998). Leiomyomas of the uterine cervix: A study of frequency. Int. J. Gynecol. Pathol..

[6] Ciravolo G., Ferrari F., Zizioli V., Donarini P., Forte S., Sartori E., Odicino F. (2019). Laparoscopic management of a large urethral leiomyoma. Int. Urogynecol. J..

[7] Ferrari F., Forte S., Valenti G., Ardighieri L., Barra F., Esposito V., Sartori E., Odicino F. (2021). Current Treatment Options for Cervical Leiomyomas: A Systematic Review of Literature. Medicina.

[8] Goia M., Disanto M.G., Ferraioli D., Palicelli A., Mitidieri M., Danese S., Picardo E. (2021). Uterine smooth muscle tumor of uncertain malignant potential. Uterine Fibroids from Diagnosis to Treatment.

[9] Soleymani Majd H., Ferrari F., Gubbala K., Campanile R.G., Tozzi R. (2015). Latest developments and techniques in gynaecological oncology surgery. Curr. Opin. Obstet. Gynecol..

[10] Hu J., Tao X., Yin L., Shi Y. (2016). Successful conservative treatment of cervical pregnancy with uterine artery embolization followed by curettage: A report of 19 cases. BJOG Int. J. Obstet. Gynaecol..

[11] Ko J.S., Suh C.H., Huang H., Zhuo H., Harmanli O., Zhang Y. (2021). Association of Race/Ethnicity with Surgical Route and Perioperative Outcomes of Hysterectomy for Leiomyomas. J. Minim. Invasive Gynecol..

[12] Wilke S., Benson J., Roller L. (2022). Uterine lipoleiomyoma: Case report and review of the literature. Radiol. Case Rep..

[13] Di Spiezio Sardo A., Gencarelli A., Vieira M.D.C., Riemma G., De Simone T., Carugno J. (2020). Differentiating a rare uterine lipoleiomyoma from uterine perforation at hysteroscopy: A scary story. J. Minim. Invasive Gynecol..

[14] Chandawale S.S., Karia K.M., Agrawal N.S., Patil A.A., Shetty A.B., Kaur M. (2018). Uterine lipoleiomyoma and lipoma: A rare unique case report with review of literature. Int. J. Appl. Basic Med. Res..

[15] Akbulut M., Gündoğan M., Yörükoğlu A. (2014). Clinical and pathological features of lipoleiomyoma of the uterine corpus: A review of 76 cases. Balk. Med. J..

[16] Wang X., Kumar D., Seidman J.D. (2006). Uterine lipoleiomyomas: A clinicopathologic study of 50 cases. Int. J. Gynecol. Pathol..

[17] Bolat F., Kayaselçuk F., Canpolat T., Erkanli S., Tuncer I. (2007). Histogenesis of lipomatous component in uterine lipoleiomyomas. Turk. J. Pathol..

[18] Mihmanlı V., Atik A.E. (2020). Cervical Lipoleiomyoma: Case Report. J. Acad. Res. Med..

[19] Agrawal P., Agrawal R., Lobo A. (2020). A case of large cervical lipoleiomyoma simulating malignancy: An intraoperative dilemma. Int. J. Reprod. Contracept. Obstet. Gynecol..

[20] Ravikanth R., Kamalasekar K. (2020). A Rare Presentation of Cervical Lipoleiomyoma with Hematometra. Gynecol. Minim. Invasive Ther..

[21] Rathore R., Anand P., Butti A.K., Sharma R., Sarin N. (2018). Giant endocervical lipoleiomyoma of cervix in a young female, presenting with prolapse—An unusual presentation. Int. J. Curr. Res..

[22] Bannur H., Suranagi V., Davanageri R. (2017). Subserosal lipoleiomyoma of the cervix in a postmenopausal woman: A rare case report. J. Sci. Soc..

[23] Şengiz Erhan S., Hallaç Keser S., Soylu Boy F.N., Çom C. (2017). Lipoleiomyoma of the Uterine Cervix: A Case Report. Okmeydanı Tıp Derg..

[24] Ye X., Xu R., Yan L., Xu X., Chen X. (2016). Comparative analysis of ultrasonographic and pathologic findings of uterine lipoleiomyoma. Chin. J. Interv Imaging Ther..

[25] Adaikkalam J. (2016). Lipoleiomyoma of Cervix. J. Clin. Diagn. Res..

[26] Terada T. (2015). Giant subserosal lipoleiomyomas of the uterine cervix and corpus: A report of 2 cases. Appl. Immunohistochem. Mol. Morphol..

[27] Singh S., Nagarajan K., Srinivasamurthy B.C., Jena S.K., Sasmal P.K. (2015). Giant Plexiform Lipoleiomyoma of the Broad Ligament with Extensive Cystic Degeneration in a Reproductive-Age Female. J. Gynecol. Surg..

[28] Barnard E.P., Bakkum-Gamez J.N., Hopkins M.R., Occhino J.A. (2015). Cervical Lipoleiomyoma: Transvaginal Approach for Excision. J. Gynecol. Surg..

[29] El-Agwany A.S. (2015). Lipoleiomyoma of the uterine cervix: An unusual variant of uterine leiomyoma. Egypt. J. Radiol. Nucl. Med..

[30] Sharma S., Ahluwalia C., Mandal A.K. (2015). A rare incidental case of lipoleiomyoma cervix. Asian Pac. J. Health Sci..

[31] Mandal R., Mondal K., Pramanik P. (2015). Gigantic lipoleiomyoma of cervix with extensive hyaline degeneration: A case report with review of literature. Ann. Pathol. Lab. Med..

[32] Kalyankar V., Kalyankar B. (2014). Rare case of cervical lipoleiomyoma. J. Evol. Med. Dent. Sci..

[33] Goyal P., Agrawal D., Ghosh S., Sehgal S., Kumar A., Singh S. (2014). Giant lipoleiomyoma of the cervix mimicking ovarian cancer in a premenopausal woman: A case report and literature Review. J. Gynecol. Surg..

[34] Chaudhari J., Kothari K., Gupta A.S., Dwivedi J. (2014). Cervical Lipoleiomyoma. J. Postgrad. Gynecol. Obstet..

[35] Fagouri H., Hafidi M.R., Guelzim K., Hakimi I., Kouach J., Moussaoui D.R., Dehayni M. (2014). Lipoleiomyoma of the uterine cervix (about an observation). Int. J. Sci. Technol. Res..

[36] Agrawal D., Fotedar S., Daral R., Kumar K. (2014). Cervical lipoleiomyoma in premenopausal woman: A rare. Ann. Pathol. Lab. Med..

[37] Terada T. (2011). Huge lipoleiomyoma of the uterine cervix. Arch. Gynecol. Obstet..

[38] Walid M.S., Heaton R.L. (2010). Case report of a cervical lipoleiomyoma with an incidentally discovered ovarian granulosa cell tumor—Imaging and minimal-invasive surgical procedure. Ger. Med. Sci..

[39] Mohan H., Bhutani A., Punia R.P.S. (2002). Lipoleiomyoma of uterus: Report of 8 cases. J. Obstet. Gynaecol. India.

[40] Shintaku M. (1996). Lipoleiomyomatous tumors of the uterus: A heterogeneous group? Histopathological study of five cases. Pathol. Int..

[41] Volpe R., Canzonieri V., Gloghini A., Carbone A. (1992). “Lipoleiomyoma with metaplastic cartilage” (benign mesenchymoma) of the uterine cervix. Pathol. Res. Pract..

[42] Kim Y.S., Lee J.H. (2022). A case report of pelviscopic resection of lipoleiomyoma originating from the uterine cervix in a postmenopausal woman. Medicine.

[43] Doldan A., Otis C.N., Pantanowitz L. (2009). Adipose Tissue: A Normal Constituent of the Uterine Cervical Stroma. Int. J. Gynecol. Pathol..

[44] de Lima M.A., Pertence A.P., de Souza M.A. (1998). Heterotopic adipose tissue in the uterine cervix. Rev. Hosp. Clin. Fac. Med. Sao Paulo.

[45] Takeuchi K., Murata K., Funaki K., Fujita I., Hayakawa Y., Kitazawa S. (2000). Liposarcoma of the uterine cervix: Case report. Eur. J. Gynaecol. Oncol..

[46] Fadare O. (2006). Uncommon sarcomas of the uterine cervix: A review of selected entities. Diagn Pathol..

[47] Tandon B., Hagemann I.S., Maluf H.M., Pfeifer J.D., Al-Kateb H. (2017). Association of Li-Fraumeni Syndrome With Small Cell Carcinoma of the Ovary, Hypercalcemic Type and Concurrent Pleomorphic Liposarcoma of the Cervix. Int. J. Gynecol. Pathol..

[48] Zaman M.U., Fatima N., Memon W.A., Zaman A., Zaman S. (2019). Pure Uterine Lipoma on 18FDG PET/CT: Rare But Easy to Diagnose. Cureus.

[49] Alfarra K.S., Aldhamer A.A., Aldubaib H.S., Majoun M.A., Alrammah A.S., Alshehri F.S., Mughallis H.M., Almalki A.J., Basakran G.G., Alayed A.M. (2021). Pure Uterine Lipoma: A Report of a Rare Entity. Cureus.

[50] Furuta T., Nakai Y., Gonoi W., Kurokawa R., Okimoto N., Sakamoto N., Fukuchi H., Kobayashi H., Makise N., Abe O. (2021). Fat-forming solitary fibrous tumor of the sacrum: A case report and literature review. Radiol. Case Rep..

[51] Devins K.M., Young R.H., Croce S., Burandt E., Bennett J.A., Pesci A., Zannoni G.F., Ip P.P.C., Nielsen G.P., Oliva E. (2022). Solitary Fibrous Tumors of the Female Genital Tract: A Study of 27 Cases Emphasizing Nonvulvar Locations, Variant Histology, and Prognostic Factors. Am. J. Surg. Pathol..

[52] Ardighieri L., Palicelli A., Ferrari F., Ragnoli M., Ghini I., Bugatti M., Bercich L., Sartori E., Odicino F.E. (2022). Risk Assessment in Solitary Fibrous Tumor of the Uterine Corpus: Report of a Case and Systematic Review of the Literature. Int. J. Surg. Pathol..

[53] Hsiao S.-M., Lin H.-H., Peng F.-S., Jen P.-J., Hsiao C.-F., Tu F.-C. (2013). Comparison of robot-assisted laparoscopic myomectomy and traditional laparoscopic myomectomy. J. Obstet. Gynaecol Res.

[54] Del Priore G., Klapper A.S., Gurshumov E., Vargas M.M., Ungar L., Smith J.R. (2010). Rescue radical trachelectomy for preservation of fertility in benign disease. Fertil. Steril..

[55] Mandato V.D., Torricelli F., Mastrofilippo V., Palicelli A., Ciarlini G., Pirillo D., Annunziata G., Aguzzoli L. (2020). Accuracy of preoperative endometrial biopsy and intraoperative frozen section in predicting the final pathological diagnosis of endometrial cancer. Surg. Oncol..

[56] Mandato V.D., Palicelli A., Torricelli F., Mastrofilippo V., Leone C., Dicarlo V., Tafuni A., Santandrea G., Annunziata G., Generali M. (2022). Should Endometrial Cancer Treatment Be Centralized?. Biology.

[57] Peker N., Gündoğan S., Şendağ F. (2017). Laparoscopic Management of Huge Cervical Myoma. J. Minim. Invasive Gynecol..

[58] Garzon-Lopez O., Garzón-Lopez F., Gomez-Ponce H., Morgan-Ortiz F. (2015). Laparoscopic Management of a Huge Retro-Cervical Myoma. J. Minim. Invasive Gynecol..

[59] Chang W.-C., Chen S., Huang S.-C., Chang D.-Y., Chou L.-Y., Sheu B.-C. (2010). Strategy of cervical myomectomy under laparoscopy. Fertil. Steril..

[60] Fletcher H., Frederick J., Hardie M., Simeon D. (1996). A Randomized Comparison of Vasopressin and Tourniquet as Hemostatic Agents during Myomectomy. Obstet. Gynecol..

[61] Liu W.-M., Wang P.-H., Chou C.-S., Tang W.-L., Wang I.-T., Tzeng C.-R. (2007). Efficacy of combined laparoscopic uterine artery occlusion and myomectomy via minilaparotomy in the treatment of recurrent uterine myomas. Fertil. Steril..

[62] Cheng Z., Yang W., Dai H., Hu L., Qu X., Kang L. (2008). Laparoscopic Uterine Artery Occlusion Combined with Myomectomy for Uterine Myomas. J. Minim. Invasive Gynecol..

[63] Mittal K.R., Chen F., Wei J.J., Rijhvani K., Kurvathi R., Streck D., Dermody J., Toruner G.A. (2009). Molecular and immunohistochemical evidence for the origin of uterine leiomyosarcomas from associated leiomyoma and symplastic leiomyoma-like areas. Mod. Pathol..

[64] Kilpatrick C.C., Adler M.T., Chohan L. (2010). Vaginal Myomectomy in Pregnancy: A Report of Two Cases. South. Med. J..

[65] Takeda A., Koyama K., Imoto S., Mori M., Nakano T., Nakamura H. (2011). Temporary endovascular balloon occlusion of the bilateral internal iliac arteries to control hemorrhage during laparoscopic-assisted vaginal hysterectomy for cervical myoma. Eur. J. Obstet. Gynecol. Reprod. Biol..

[66] Diakosavvas M., Angelou K., Fasoulakis Z., Kathopoulis N., Zacharakis D., Blontzos N., Antsaklis P., Haidopoulos D., Daskalakis G., Rodolakis A. (2022). Myomectomy during pregnancy; diagnostical dilemmas: Two case reports and a systematic review of the literature. J. Obstet. Gynaecol..

[67] Krzyzanowski J., Wozniak S., Szkodziak P., Krzyzanowski A., Wojciech W., Paszkowski T. (2022). Minimally invasive treatment options for uterine fibroids—State-of-the art 2021. Ginekol. Pol..

[68] Cavaliere A.F., Vidiri A., Gueli Alletti S., Fagotti A., La Milia M.C., Perossini S., Restaino S., Vizzielli G., Lanzone A., Scambia G. (2021). Surgical Treatment of “Large Uterine Masses” in Pregnancy: A Single-Center Experience. Int. J. Environ. Res. Public Health..

[69] Yang Y., Yang Y., You M., Chen L., Sun F. (2022). Observation of pregnancy outcomes in patients with hysteroscopic resection on submucous myomas. J. Obstet. Gynaecol. Res..

[70] Keriakos R., Maher M. (2013). Management of Cervical Fibroid during the Reproductive Period. Case Rep. Obstet. Gynecol..

[71] Palicelli A., Giaccherini L., Zanelli M., Bonasoni M., Gelli M., Bisagni A., Zanetti E., De Marco L., Torricelli F., Manzotti G. (2021). How Can We Treat Vulvar Carcinoma in Pregnancy? A Systematic Review of the Literature. Cancers.

[72] D’Agostino C., Surico D., Monga G., Palicelli A. (2019). Pregnancy-related decidualization of subcutaneous endometriosis occurring in a post-caesarean section scar: Case study and review of the literature. Pathol.-Res. Pract..

[73] Higuchi Y., Okuda K., Nakamura Y., Hayashi A., Hayashi M., Fujiyama F., Yoshida Y., Yamashita Y., Terai Y., Kamegai H. (2012). Efficacy and safety of bipolar electrode grasping forceps for laparoscopic myomectomy in uterine cervical myoma. Asian J. Endosc. Surg..

[74] Takeda A., Koyama K., Imoto S., Mori M., Sakai K., Nakamura H. (2009). Temporary endovascular balloon occlusion of the bilateral internal iliac arteries for control of hemorrhage during laparoscopic-assisted myomectomy in a nulligravida with a large cervical myoma. Fertil. Steril..

[75] Tian J., Hu W. (2012). Cervical leiomyomas in pregnancy: Report of 17 cases. Aust. N. Z. J. Obstet. Gynaecol..

[76] Gurung G., Rana A., Bahadur Rana Magar D. (2003). Utero-vaginal prolapse due to portio vaginal fibroma. J. Obstet. Gynaecol. Res..

[77] Oruç S., Karaer O., Kurtul O. (2004). Coexistence of a prolapsed, pedunculated cervical myoma and pregnancy complications: A case report. J. Reprod. Med..

[78] González González V., Herráez Moreta A., Mayoral Triana A., Riolobos Sierra L., Cristóbal García I., Izquierdo Méndez N. (2020). Prolapsed cervical myoma during pregnancy. Eur J. Obstet. Gynecol. Reprod. Biol..

[79] Gandhi A., Dugad H., Shah Y. (2014). A rare presentation of cervical fibroid in pregnancy. Ann. Afr. Med..

[80] Lohle P.N.M., Boekkooi P.F., Fiedeldeij C.A., Berden H.J.J.M., de Jong W., Reekers J.A., Franx A., van Rooij W.J.J. (2015). Selective Embolisation of a Heavily Bleeding Cervical Fibroid in a Pregnant Woman. Cardiovasc. Interv. Radiol..

[81] Kamra H.T., Dantkale S.S., Birla K., Sakinlawar P.W., Narkhede R.R. (2013). Myxoid leiomyoma of cervix. J. Clin. Diagn. Res..

[82] Scott R.B., Spence J.M. (1951). Delivering submucous myoma complicating pregnancy. Am J. Obstet. Gynecol..

[83] Rozanska-Waledziak A., Kacperczyk-Bartnik J., Bartnik P., Waledziak M., Czajkowski K. (2021). A successful vaginal myomectomy of cervical leiomyoma in early pregnancy. Ginekol. Pol..

[84] Holub Z., Mara M., Kuzel D., Jabor A., Maskova J., Eim J. (2008). Pregnancy outcomes after uterine artery occlusion: Prospective multicentric study. Fertil. Steril..

[85] DeMeritt J.S., Wajswol E., Wattamwar A. (2019). Pregnancy after Superselective Embolization of the Cervicovaginal Arteries for a Bleeding Cervical Fibroid. J. Vasc. Interv. Radiol..

[86] Van der Meulen J.F., Pijnenborg J.M., Boomsma C.M., Verberg M.F., Geomini P.M., Bongers M.Y. (2016). Parasitic myoma after laparoscopic morcellation: A systematic review of the literature. BJOG Int. J. Obstet. Gynaecol..

[87] Leren V., Langebrekke A., Qvigstad E. (2012). Parasitic leiomyomas after laparoscopic surgery with morcellation. Acta Obstet. Gynecol. Scand..

[88] Ghorbani H., Ranaee M., Vosough Z. (2020). Two Rare Cases of Uterine Leiomyosarcomas Originating from Submucosal Leiomyomas Proved by Their Immunohistochemistry Profiles. Int. J. Fertil. Steril..

[89] Yamaguchi M., Kusunoki S., Hirayama T., Fujino K., Terao Y., Itakura A. (2019). Case of leiomyosarcoma arising from subserosal leiomyoma. J. Obstet. Gynaecol. Res..

[90] McDonald A.G., Dal Cin P., Ganguly A., Campbell S., Imai Y., Rosenberg A.E., Oliva E. (2011). Liposarcoma arising in uterine lipoleiomyoma: A report of 3 cases and review of the literature. Am. J. Surg. Pathol..

[91] U.S. Food and Drug Administration (2020). Update: Perform Only Contained Morcellation When Laparoscopic Power Morcellation Is Appropriate.

[92] Mandato V.D., Torricelli F., Pirillo D., Aguzzoli L., Abrate M., Palomba S., La Sala G.B. (2016). Impact of the Food and Drug Administration Safety Communication on the Use of Power Morcellator in Daily Clinical Practice: An Italian Survey. J. Minim. Invasive Gynecol..

[93] Ameer M.A., Fagan S.E., Sosa-Stanley J.N., Peterson D.C. (2021). Anatomy, Abdomen and Pelvis, Uterus.

[94] Chen C.J., Thompson H. (2021). Uterine Prolapse.

[95] Jeon M.J. (2019). Surgical decision making for symptomatic pelvic organ prolapse: Evidence-based approach. Obstet. Gynecol. Sci..

[96] Doshani A., Teo R.E., Mayne C.J., Tincello D.G. (2007). Uterine prolapse. BMJ.

[97] Gutman R.E., Ford D.E., Quiroz L.H., Shippey S.H., Handa V.L. (2008). Is there a pelvic organ prolapse threshold that predicts pelvic floor symptoms?. Am J. Obstet. Gynecol..

[98] Parvathavarthini K., Vanusha A. (2019). Clinical epidemiological study of uterine prolapse. Int. J. Reprod. Contracept. Obstet. Gynecol..

[99] Carley M.E., Schaffer J. (2000). Urinary incontinence and pelvic organ prolapse in women with Marfan or Ehlers Danlos syndrome. Am J. Obstet. Gynecol..

[100] Paladini D., Di Spiezio Sardo A., Mandato V.D., Guerra G., Bifulco G., Mauriello S., Nappi C. (2007). Association of cutis laxa and genital prolapse: A case report. Int. Urogynecol. J..

[101] Onowhakpor E.A., Omo-Aghoja L.O., Akani C.I., Feyi-Waboso P. (2009). Prevalence and determinants of utero-vaginal prolapse in southern Nigeria. Niger. Med. J..

[102] Turhan N., Simavli S., Kaygusuz I., Kasap B. (2014). Totally inverted cervix due to a huge prolapsed cervical myoma simulating chronic non-puerperal uterine inversion. Int. J. Surg. Case Rep..

[103] Abe S. (1961). Prolapse of uterus caused by myoma of the uterus with cervical tumor. Case reports. J. Jpn. Obstet. Gynecol. Soc..

[104] Ben-Baruch G., Schiff E., Menashe Y., Menczer J. (1988). Immediate and late outcome of vaginal myomectomy for prolapsed pedunculated submucous myoma. Obstet. Gynecol..

[105] Okonkwo J.E.N., Obiechina N.J.A., Obionu N. (2003). Incidence of pelvic organ prolapse in nigerian women. J. Natl. Med. Assoc..

[106] Milsom I., Altman D., Cartwright R., Lapitan M.C., Nelson R., Sjöström S., Tikkinen K.A.O., Abrams P., Cardozo L., Wagg A., Wein A. (2017). Epidemiology of urinary incontinence (UI) and other lower urinary tract symptoms (LUTS), pelvic organ prolapse (POP) and anal (AI) incontinence. Incontinence.

